# Rice protein-binding microarrays: a tool to detect cis-acting elements near promoter regions in rice

**DOI:** 10.1007/s00425-021-03572-w

**Published:** 2021-01-21

**Authors:** Joung Sug Kim, SongHwa Chae, Kyong Mi Jun, Gang-Seob Lee, Jong-Seong Jeon, Kyung Do Kim, Yeon-Ki Kim

**Affiliations:** 1 Department of Biosciences and Bioinformatics, Myongji University, 116 Myongji-ro, Cheoin-gu, Yongin, Gyeonggi-do 17060 Republic of Korea; 2 Genomics Genetics Institute, GreenGene BioTech Inc., 16-4 Dongbaekjungang-ro 16beon-gil, Giheung-gu, Yongin, Gyeonggi-do 17015 Republic of Korea; 3 Department of Agricultural Biotechnology, National Institute of Agricultural Sciences, Jeonju, 54875 Republic of Korea; 4 Graduate School of Biotechnology and Crop Biotech Institute, Kyung Hee University, Yongin, Gyeonggi-do, 17104 Republic of Korea

**Keywords:** Transcription factors, Protein-binding microarray, DNA-binding sequence, *OsWOX13*, *OsSMF1*, *OsWRKY34*

## Abstract

**Main conclusion:**

The present study showed that a rice (*Oryza sativa*)-specific protein-binding microarray (RPBM) can be applied to analyze DNA-binding motifs with a TF where binding is evaluated in extended natural promoter regions. The analysis may facilitate identifying TFs and their downstream genes and constructing gene networks through cis-elements.

**Abstract:**

Transcription factors (TFs) regulate gene expression at the transcriptional level by binding a specific DNA sequence. Thus, predicting the DNA-binding motifs of TFs is one of the most important areas in the functional analysis of TFs in the postgenomic era. Although many methods have been developed to address this challenge, many TFs still have unknown DNA-binding motifs. In this study, we designed RPBM with 40-bp probes and 20-bp of overlap, yielding 49 probes spanning the 1-kb upstream region before the translation start site of each gene in the entire genome. To confirm the efficiency of RPBM technology, we selected two previously studied TFs, *OsWOX13* and *OsSMF1*, and an uncharacterized TF, *OsWRKY34*. We identified the ATTGATTG and CCACGTCA DNA-binding sequences of OsWOX13 and OsSMF1, respectively. In total, 635 and 932 putative feature genes were identified for *OsWOX13* and *OsSMF1*, respectively. We discovered the CGTTGACTTT DNA-binding sequence and 195 putative feature genes of *OsWRKY34.* RPBM could be applicable in the analysis of DNA-binding motifs for TFs where binding is evaluated in the promoter and 5′ upstream CDS regions. The analysis may facilitate identifying TFs and their downstream genes and constructing gene networks through cis-elements.

**Supplementary Information:**

The online version contains supplementary material available at 10.1007/s00425-021-03572-w.

## Introduction

In eukaryotic organisms, gene expression is controlled by the interactions of DNA elements known as cis-elements and regulatory proteins according to their implementation of genetic information during development or during responses to an external stimulus (Srivastava et al. 2018; Zou et al. 2011). The basic expression mechanisms are also exerted in plants, and DNA elements are specified as promoters, enhancers, silencers and insulators. The promoters are located close to the transcription start sites and initiate transcription by providing RNA polymerase II binding sites or sites for other regulatory proteins. Other elements are generally present distally or proximally to the promoters and, in many cases, are found in untranslated regions of mRNAs. These elements are involved in various mRNA properties such as translation efficiency and stability, which are also important in the posttranscriptional control of transcripts. Transcription factors (TFs) play a pivotal role in regulating gene expression by binding to their cognate motifs in promoter regions.

The interaction between cis-elements and TFs has traditionally been performed by biochemical assays such as electrophoretic mobility shift assays (EMSAs), nitrocellulose filter-binding assays, footprinting assays, and yeast one-hybrid system assays (Hellman and Fried 2007; Helwa and Hoheisel 2010). Although these assays are pivotal to identify cis-elements, some approaches are still laborious and slow, and many TFs remain uncharacterized. Recent high-throughput methods such as chromatin immunoprecipitation (ChIP)-chip, ChIP followed by sequencing (ChIP-seq), DNA–protein interaction enzyme-linked immunosorbent assay (DPI-ELISA), and protein-binding microarrays (PBM) have been developed with the availability of whole-genome sequences and advances in microarray technology (Barski et al. 2007; Brand et al. 2010; Ren et al. 2000; van Steensel et al. 2001; Wang et al. 2008).

PBMs were introduced to conveniently determine protein–DNA interactions in vitro (Berger and Bulyk 2009). In this technology, the carefully designed sequences of single-stranded DNA are synthesized on microarrays, and then the complementary DNA strand is synthesized with DNA polymerase in the presence of dNTPs. The interactions between DNA sequences and TFs are detected by labeling antibodies against TFs or fluorescence TFs depending on the technologies. The sequence designs for PBMs were improved by adapting de Bruijn sequences and the in situ synthesis of DNA oligonucleotides on slides (Berger et al. 2006). In the design, all possible DNA sequence variants of a given length k were applied on a single, universal microarray; thus, all k-mer microarrays covering all 10-base pair (bp) binding sites were designed. With the genome era, a genome-mimicking PBM, particularly in yeast, was also prepared by spotting double-stranded DNA (Zhu et al. 2009). Recently, a custom PBM was developed in an effort to characterize the DNA-binding activity of transcription activator-like effectors (TALEs), which are secreted by the bacteria Xanthomonas via their Type III secretion system function and function as virulence factors (Anderson et al. 2020; Rogers et al. 2015). TALE–DNA interactions were comprehensively assayed in this PBM in which ~ 5000–20,000 unique DNA sequences per effector protein were spotted.

The identification of genomic regulatory elements also led to the construction of the databases TRANSFAC (Wingender et al. 1996), GRASSIUS (Yilmaz et al. 2009), PlnTFDB (Perez-Rodriguez et al. 2010), UniPROBE (Hume et al. 2015), and PlantTFDB (Jin et al. 2017). In particular, PlantTFDB was constructed based on a collection of 156 plant species with sequenced genomes. Recent advances in ChIP-seq have provided powerful ways to identify genome-wide profiling of DNA-binding proteins and histone modifications, leading to databases such as ChEA, CistromeMap, and ChIPBase (Lachmann et al. 2010; Qin et al. 2012; Yang et al. 2013).

Kim et al (2009) designed a PBM, denoted Q9-PBM, in which the feature probes are quadruples of all possible 9-mer combinations (Kim et al. 2009). In the initial design of all possible 4^9-mers (262,144 reads), 131,072 features that denote DNA segments corresponding to a physical position on a microarray were selected after considering the reverse complementary sequences because double-stranded DNA has a bidirectional aspect. The quadruple sequences might offer several advantages, such as increasing the binding chances or satisfying the repeat requirements of the TFs. Q9-PBM employs DsRed fluorescent protein, which eliminates multiple wash and hybridization steps. Q9-PBM confirmed the well-known DNA-binding sequences of Cbf1 and CBF1/DREB1B, and it was also applied to elucidate the unidentified cis-acting elements of the OsNAC6, MYB44, and OsSMF1 rice TFs (Kim et al. 2009). These PBMs can identify binding motifs but are limited by the number of designed nucleotide sequences in terms of oligomer length (9 or 10). The binding sites of TFs may also be searched using gene-specific promoters on microarrays.

To identify the binding motif for TFs in rice using the minimum number of feature probes and to investigate the binding activity in the promoter regions in rice, we designed a rice PBM (RPBM) such that overlapping 40-nt probes covered the 1-kb gene-specific upstream region. The single oligomers on the microarray were subjected to polymerase chain reaction (PCR) to form double strands, and then the binding sites of the TFs OsWOX13 and OsSMF1 were tested. OsWOX13 preferentially binds to an ATTGATTG DNA-binding motif, while OsSMF1 has multiple DNA-binding motifs such as GCN4 [TGA(G/C)TCA], ACGT [CCACGT(C/G)], and ATGA [GGATGAC] (Kim et al. 2017; Minh-Thu et al. 2018). Using this RPBM, not only were the DNA-binding motifs and known putative target genes OsWOX13 and OsSMF1 identified but the RPBM was also applied to identify those of an uncharacterized TF, OsWRKY34.

## Materials and methods

### Protein expression and purification

All the proteins used in this study were expressed as N-terminal fusions to a polyhistidine-tag and the DsRed fluorescent protein. The coding sequence of the DsRed fluorescent protein was amplified from the pDsRed monomer vector (Clontech) by PCR and inserted into the pET32a expression vector (Novagen). Full-length *OsWOX13* (Os01g60270, F: GGGATATCATGGAGTGGGACAAGG, R: TTGCGGCCGCCATACATATC AAAGCTTTCACC), *OsSMF1* (Os07g0182000, F: GGGATATCATGGAGCACGTGTTCGC, R: GGGAATTCCTACTGAAGCTCCATGTTGA) and *OsWRKY34* (Os04g0545000, F: GGGATATCATGTATGCGTGCATGGAAGG, R: TTGCGGCCGCCGAAGGAGGTGAAGGCGCA were amplified from the cDNA of *O. sativa* and inserted into the pET32a-DsRed recombinant vector. These proteins were expressed in the *Escherichia coli* strain BL21-CodonPlus (Stratagene). The overnight-cultured cells were inoculated in fresh liquid LB media, grown at 37 °C to an OD_260_ of 0.6 and induced with 1 mM isopropyl β-d-1-thiogalactopyranoside (IPTG) at 25 °C for 5 h. Cell pellets were resuspended in 5 ml of phosphate-buffered saline (PBS) buffer including protease inhibitor and sonicated to lysis for 5 min at 45-s intervals on ice. The supernatant soluble fractions were retained after centrifugation at 4 °C for 20 min at 14,000*g*. The proteins were enriched using Ni–NTA resins (Stratagene) according to the manufacturer’s protocols. The purified protein fractions were collected in a volume of 500 µl, and concentrations were determined.

### Synthesis of complementary strands on microarrays

Complementary DNA strands were synthesized as described in a previous report (Kim et al. 2009, 2012). The reaction solution contained 40 µM dNTPs (TaKaRa), 1.6 µM CyDye5-dUTP (GE Healthcare), 1 µM 5′-CTGCACTAGGTGACTCCG-3′ primer (Bioneer), 1 × ThermoSequenase buffer, and 0.5 U/µl of ThermoSequenase (USB). A custom-designed PBM (Agilent) was combined with the reaction solution in a hybridization chamber (Agilent) according to the manufacturer’s protocol. The assembled hybridization chamber was incubated at 85 °C for 10 min and then 60 °C for 90 min. The microarray was washed in PBS-0.01% (v/v) Triton X-100 at 37 °C for 1 min, PBS-0.01% (v/v) Triton X-100 at 37 °C for 10 min, and PBS at room temperature for 3 min, followed by drying by centrifugation at 500 g for 2 min. The doubled-stranded microarray was scanned using a 4000B microarray scanner (Axon) to verify successful synthesis.

### Protein-binding microarray

Double-stranded microarrays were washed with PBS containing 0.01% (v/v) Triton X-100 and blocked with PBS containing 2% (wt/v) BSA (Sigma) for 1 h. Next, the microarray was first washed with PBS containing 0.1% (v/v) Tween-20 and then with PBS containing 0.01% (v/v) Triton X-100 for 1 min. The protein-binding mixture was prepared containing 200 nM TF in PBS containing 2% (wt/v) BSA, 51.3 ng/µl of salmon testes DNA (Sigma, D1626), and 50 µM zinc acetate. The prepared protein mixture was incubated to stabilize and bind the microarray at 25 °C for 1 h. The microarray was first washed for 2 min with PBS containing 50 µM zinc acetate and 0.5% (v/v) Tween-20 for 10 min and then with PBS containing 50 µM zinc acetate and 0.01% Triton X-100 for 2 min, and finally with PBS containing 50 µM zinc acetate. Fluorescence images were obtained using a 4000B microarray scanner (Axon). Each microarray was scanned three to five times at full laser power intensity and a pixel resolution of 5 mm. To obtain 0.01–0.05% (20–100 spots) of Cy3-saturated spots, different photomultiplier tube (PMT) gain settings were applied, ranging from 550 to 780 for Cy3 intensity. The microarray was rescanned whenever the number of saturated spots was not in this range. However, the maximum Cy5 PMT gain setting was used to identify the spot positions.

### Analysis of the transcription factor motifs

To extract the transcription factor-binding motifs, the signal intensities of feature probes were chosen for those with intensities higher than background. When the intensities of the features (approximately 950,000) were rank ordered and depicted on the *x*–*y* coordinates, a deep left slope followed by a heavy right tail was observed. Two *y* = *ax* + *b* models were applied to a steep left region and a tail right region with R (https://www.r-project.org/), respectively. An extrapolated y intercept was obtained from the line of the tail region and was used to choose the significant binding features.

To determine the best binding motif, the 40-bp feature sequences are split into 5–11 k-mers with a base shift and the feature intensities are assigned to those oligomers. For example, 36,790 feature probes of PBM with the OsWOX13 transcription factor from the steep region were split into 9-mers with a base shift, and the intensities of the feature were applied to the oligomer as an initial intensity. Thus, the feature probes produced 32 9-mers. The features from the steep region produced 198,384 distinct 9-mers and the total occurrence was 1,177,287. We found 4–5 consecutive G- or C-rich oligomers (3148) with nonspecific binding with all the TFs tested in this report and discarded them from subsequent analysis. Each transcription factor has a unique distribution of intensity and the occurrence of 9-mers. TFs such as OsWOX13 and OsWRKY34 were sorted according to the intensity, while OsSMF1 was sorted according to the product of the intensity and occurrence. To narrow the motifs of OsWOX13, clusters were formed allowing 2 mismatches, with a 5-nt sequence matching the template of the highest intensity and with 1028 oligomers clustered with GATTGATTG as a seed. A sequence logo was generated using weblogo (weblogo.berkeley.edu) for the cluster.

OsSMF1 also provides a similar rank-ordered signal distribution showing a deep left slope followed by a heavy right tail. To identify the binding motif of OsSMF1, a 40-bp probe is split into 9-mers, and each oligomer gives the pseudo intensity of the probe as with OsWOX13. The 15,394 probes produce 178,857 distinct oligomers, and the total occurrence is 492,608. We found 4–5 consecutive G- or C-rich oligomers (16,853) with nonspecific binding and discarded them from subsequent analysis. The 9-mer GCCACGTCA (834) was the most frequent. Three additional clusters were obtained—ACGTAAGCG, TGAGTCA, and GGATGAC—and the number of members was 24, 43 and 24, respectively. Web logo analysis for the ACGTAAGCG members suggest they are part of GCCACGT(c/a)AG.

### Positional mutation effects of the motif based on the signal intensity

To test the significance of the base of the motif, the signal intensities of the point-mutated oligomers at each position were summed, averaged and compared with the wild-type motif. The signal intensities around the motif were also tested such that 1 base was extended either in the 5′ or 3′ direction. The oligomer with the highest intensity among those with the four different bases at each direction was chosen as the base-extended oligomer. For example, G was extended in the 5′ direction in the case of ATTGATTG, resulting in the same-sized oligomer—GATTGATT among AATTGATT, TATTGATT, CATTGATT and GATTGATT. The highest signal intensity of the oligomer at two bases ahead was searched from the one base-extended oligomer. Similarly, positional mutation analysis of the oligomers ranging from 5- to 10-mers was also analyzed.

### Selection of cofound motifs with ATTGATTG motifs

The 1-kb promoter regions of 29,379 rice genes were retrieved from RAP-DB (rapdb.dna.affrc.go.jp/). The genes containing ATTGATTG were selected using an in-house Perl script. In total, 1631 genes contained the motif in their promoters. Promoter regions 1-kb long were also retrieved from the same database. To identify cis-elements and TFs that might be associated with OsWOX13, the TFs and their associated cis-elements of *Oryza sativa* were downloaded from the Plant Transcription Factor Database (Jin et al. 2017; http://planttfdb.gao-lab.org/). The representative cis-elements were extracted under the following criteria: first, the nucleotides with higher occupancies than 0.5 at each position in the letter-probability matrix were extracted; second, the motifs with at least 6 distinctive nucleotides and nonconsecutive Ns were chosen for further analysis. The genes in the upstream 1-kb regions were searched for representative sequences.

### Electrophoretic mobility shift assay (EMSA)

First, 5′ FAM-end labeled and unlabeled oligonucleotides were annealed with each complementary sequence. Five micrograms of OsWOX13 and OsSMF1 protein was incubated with 40 fmol of FAM-labeled double-stranded oligonucleotides, 1 μg of poly dI-dC, 1X binding buffer, 2.5% (v/v) glycerol and 0.05% (wt/v) NP-40 in a 20-μl reaction volume for 1 h at room temperature according to the manufacturer’s instructions (Pierce). The reaction mixture was then analyzed by electrophoresis in a nondenaturing 6% acrylamide gel with 0.5X TBE buffer. The DNA–protein complexes in the gel were detected as fluorescence signals using Fusion SL (Vilber Lourmat).

### Statistical analysis of the motifs

A position matrix for a clustered sequences of each transcription factor was formed, and 4 bases at each position were counted using an in-house Perl script. For example, a matrix suggested an 8-mer, ATTGATTG might be a binding motif of OsWOX13. The significance of the motif was tested by the Wilcoxon–Mann–Whitney test in R-language. The ranks of the features containing the motif from the file sorted according to their signal intensities were compared with those of the features without the motif. Similarly, the intensities and ranks of the feature probes were also analyzed with 5-, 6-, 7-, 8-, 10-, and 11-mers.

## Results

### Design of the RPBM

**Fig. 1:**
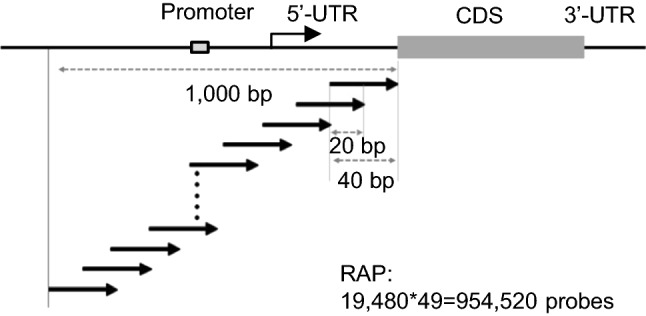
Schematic of the rice promoter protein-binding microarray. The probe is 40 bp long, of which 20 bp overlaps. For each gene, 49 probes spanned the 1-kb promoter region before the translation start site. In total, 954,520 probes were designed from 19,480 genes among 31,439 genes. The promoter and transcriptional start positions are conceptually denoted

Probes for the RPBM were designed from promoters of genes deposited in the IRGSP RAP3 database (rapdb.dna.affrc.go.jp/). A feature probe is 40 bp long, covering a gene-specific region, with 20 bp for an annealing site for PCR. Each gene-specific region overlapped 20 bp, and the corresponding 49 feature probes spanned the 1-kb upstream region before the translation start site of each gene (Fig. 1). Considering the ambiguity of annotation, the first feature probe was designed from the translational start site for a transcript without a 5′-UTR (3822) or from 200 bp 3′ upstream region of the transcriptional start site for a transcript with a 5′-UTR longer than 200 bp (4301). Thus, 954,520 probes were designed from 19,480 genes among 29,379 genes. Each feature was followed by a primer linker sequence (5′-CGGAGTCACCTAGTGCAG-3′) and a 5-nt thymidine linker (TTTTT) on the microarray.

### Analysis of signal intensities

The full-length *OsWOX13*,* OsSMF1*,* and OsWRKY34* cDNAs were fused at the N-terminus of the *DsRed* fluorescent protein gene and hybridized to the RPBM as described in the Methods section. Next, the consensus-binding motifs were determined based on the signal strength (Kim et al. 2009, 2017).

A rank-ordered signal distribution showed a steep slope on the left, followed by a heavy right tail for RPBM. The signal distribution of RPBM features with OsWOX13 was depicted (Fig. S1a). For OsWOX13, the number of features with its intensity higher than background was 889,720 and its mean intensity (± sd) was 1813 ± 4890.0 (Fig. S1b left panel). We assumed that the signal distribution was due to specific interactions between the proteins and features on the microarray. Two independent linear models, *y* = *ax* + *b*, were independently applied in the steep and heavy right tail regions, respectively, using the R statistical language. In OsWOX13, the slope and *y*-axis intercept of the steep sloping region were − 14.7 and 66,570.6, respectively, while those of the heavy tail region were − 0.0043 and 3144.4, respectively (Fig. S1a). The extrapolated value, 3144.4, from the heavy right tail regions was chosen as a cutoff for significant signal intensities for OsWOX13.

For OsSMF1, a rank-ordered signal distribution also showed a steep slope on the left, followed by a heavy right tail for RPBM (Fig. S2a). The number of features with an intensity higher than background was 940,950 and the mean intensity was 1973.4 ± 3289.6 (Fig. S2b left panel). The slope and *y*-axis intercept of the steep slope region were − 25.1 and 64,928.8, respectively, while those of the heavy tail region were − 0.0207 and 3129.9, respectively (Fig. S2a). The extrapolated value, 3120.9, from the heavy right tail regions was chosen as a cutoff for significant signal intensities for OsSMF1.

The strong binding features from the deep slope with intensities higher than 3144.4 and 3129.9 numbered 36,790 (Table S1) and 63,811 (Table S2) for OsWOX13 and OsSMF1, respectively. These results suggest that the binding of transcription factors and their cognate binding sites in RPBM were as stable as those found in Q9-PBM. In addition, the probe design from the promoter regions overcame potential complexities because of the concatemers of the feature sites.

### Identifying the DNA-binding motif and putative feature genes of OsWOX13 by RPBM

**Fig. 2:**
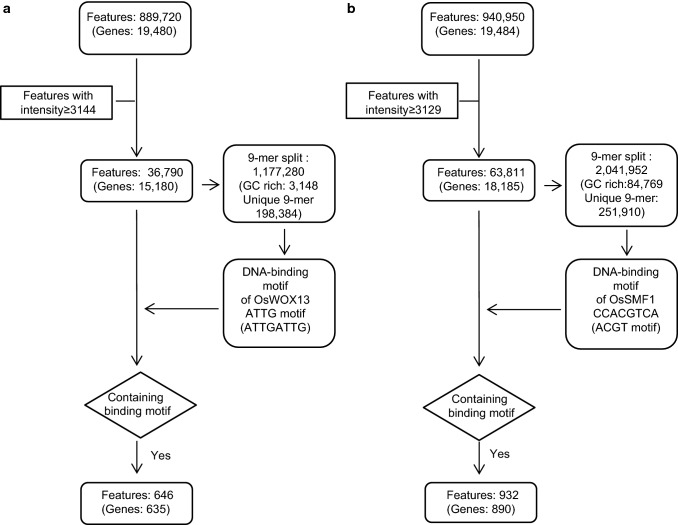
Flowchart of the process for identifying putative feature genes of transcription factors OsWOX13 (**a**) and OsSMF1 (**b**)

To identify the DNA-binding motif of OsWOX13, a 40-bp feature probe was split into 9-mers with a base shift, and the intensity of the feature probe was applied to the 9-mers; finally, the probe produced 32 9-mers (Fig. S3). The features (889,720) produced 28,471,040 distinct 9-mers with an average intensity of 1813.7. Among them, feature probes from the steep region (36,790) produced 198,384 distinct 9-mers from 1,177,280 of the total occurrence (Fig. 2a, Table S1). We found that 4–5 consecutive G- or consecutive C-rich features (3148) exhibited nonspecific binding and discarded them from subsequent analysis. The average intensity and occurrence of 9-mers from the significant signal intensities were 21,193.0 and 5.9, respectively. These 9-mers were sorted according to their pseudo-intensities, and GATTGATTG showed the highest intensity of 37,706 with an occurrence of 280 (Table S3).

**Fig. 3:**
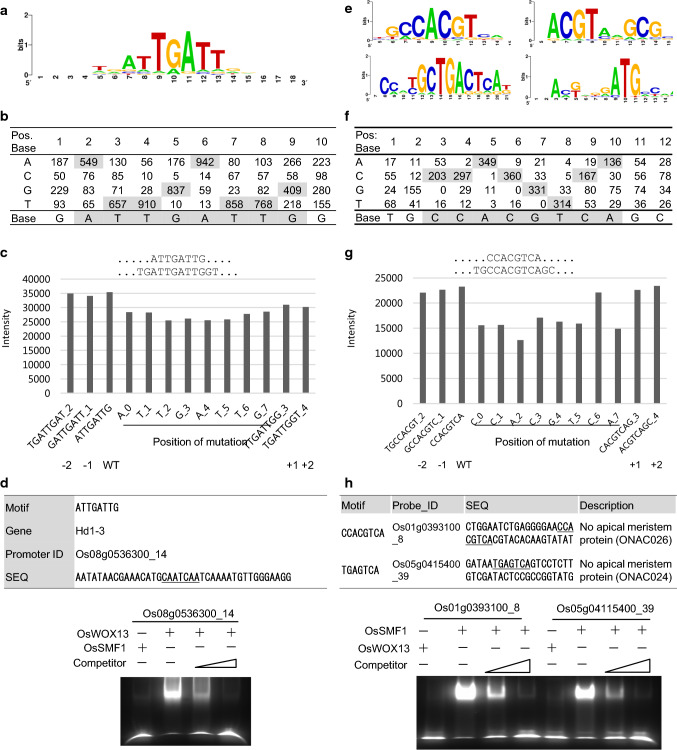
DNA-binding motif analysis of OsWOX13. **a**, **e** DNA-binding motifs of OsWOX13 and OsSMF1 determined by clustering of the significant binding sequences. They were visualized using the Web logo program (weblogo.berkeley.edu). Analysis of OsSMF1 provided at least 4 clusters; each cluster was analyzed, and its position weight matrix was calculated. **b**, **f** Position weight matrix from the clustering of 9-mers. **c**, **g** Comparison of the intensities of oligomers with point mutations at distinct positions in ATTGATTG (**c**) and GCCACGTCA (**g**). Binding motif of OsWOX13 from the Wilcoxon–Mann–Whitney test, *p* value 0. **d** EMSA-based competition analysis of OsWOX13 using the probe Os08g0536300_14, which contains the ATTGATTG motif. The 40-bp sequences used as probes and their competitors are depicted. EMSAs were performed using the OsWOX13:DsRed protein and a probe 5′-labeled with FAM. Competition for the labeled sequences was tested by adding different concentrations of unlabeled probes. **h** EMSA-based competition analysis of OsSMF1. The 40-bp sequence feature probes, Os01g0393100_8 and Os05g0415400_39, representing GCCACGT and TGAGTCA clusters, respectively, were used as probes, and competitors are depicted. EMSAs were performed using the OsSMF1:DsRed protein and a probe 5′-labeled with FAM. Competition for the labeled sequences was tested by adding different concentrations of unlabeled probes

To identify a consensus sequence, cluster analysis was performed such that 9-mer clusters were formed with an allowance of 2 mismatches and with a 5-nt sequence matching the template of the highest intensity belonging to a group as described in the Methods section. The 1,028 9-mers formed a cluster with GATTGATTG as a template. These top 20 9-mers ranked by intensity contained one or more ATTG sequences (Table 1). Web logo (weblogo.berkeley.edu) provided ATTGATTG (Fig. 3a). The occurrences of nucleotides at each position were shown in a position weight matrix (PWM) by clustering these 9-mers (Fig. 3b).

**Table 1:** List of 9-mers highly ranked by intensity and containing the ATTGATTG sequence

Rank^a^	9-Mer^b^	Intensity_ave^c^	Occurrence_total^d^	Int_ave*Occur_tot^e^	Occur_diff_pos^f^
1	GATTGATTG	37,706.65	280	10,557,862	31
2	ATTGATTGA	37,026.91	270	9,997,266	31
3	TTGATTGAT	36,995.96	343	12,689,615	31
4	TGATTGATT	36,509.81	401	14,640,432	31
5	ATTGATTGG	36,080.41	158	5,700,705	29
6	TAATTGATT	35,690.72	274	9,779,257	31
7	GATTGACAG	35,273.34	41	1,446,207	17
8	GATTGATTA	35,246.55	126	4,441,065	29
9	ATTGATTGC	35,134.35	124	4,356,660	28
10	TGATTGATG	34,743.81	183	6,358,117	30
11	GTGATTGAT	34,695.00	139	4,822,605	30
12	TGATTGGCG	34,639.79	34	1,177,753	18
13	TATTGATTG	34,572.04	95	3,284,344	23
14	CTGATTGAT	34,351.64	121	4,156,549	26
15	TGATTGATA	34,202.91	126	4,309,567	30
16	AATTGATTG	34,058.06	193	6,573,205	28
17	ATGATTGAC	33,941.15	60	2,036,469	21
18	GACTGATTG	33,741.80	35	1,180,963	17
19	GATTGATGG	33,686.23	74	2,492,781	27
20	ATTGATAGC	33,661.22	27	908,853	16

^a^Rank order by intensity

^b^9-Mers were obtained by a base shift on a 40-nt feature probe; finally, the probe produced 32 distinct 9-mers

^c^The intensities were averaged over all the feature probes containing the corresponding 9-mer sequence

^d^Total number of occurrences of the 9-mer from the 34,778 strongly binding feature probes

^e^Total intensities for column c * column d

^f^Distinct positions of the 9-mers in the 40-nt probes. The highest value (near 32) suggests that the 9-mers were obtained from all the positions by a base shift in the probes

The distribution of the ATTGATTG motif-containing features among the RPBM features was also depicted (Fig. S1b). The features containing ATTGATTG or its reverse complementary sequence, CAATCAAT, numbered 906 and 980, respectively The number of features with the forward motif ATTGATTG was 743, and the mean intensity was 30,876.2 ± 18,406.8; 163 were found in the tail regions (Fig. S1b, labeled with Steep and Tail, respectively**)**. The reverse complementary motif CAATCAAT numbered 603, and the mean intensity was 18,395.4 ± 13,756.0; 377 features were found in the tail region (Fig. S1b, labeled with Steep and Tail, respectively). In total, among 1886 features containing the element, 1345 (71.3%) were found in the strong binding zone. The number of features without the motif (None_cis_features) was 887,834, and the mean intensity was 1778.4 ± 4759.9. Thus, the signal intensities of the features containing the forward and reverse complementary motifs were 17.4- and 10-fold higher, respectively, than those of the features without the motif. In addition, the feature intensity of the forward motif was 1.7-fold higher than that of the reverse. The Wilcoxon–Mann–Whitney test was performed for the feature probes containing ATTGATTG and those without the sequence, with a *p* value of 0, suggesting that ATTGATTG contributed significantly to binding.

In addition, mutation analysis was conducted by changing bases in each ATTGATTG as described in the Methods section (Fig. 3c). A base-mutated sequence showed a maximum decrease at the 4th nt, G, and a minimum at the 1st nt, A (10,756.4 and 10,139.6, respectively). The Wilcoxon–Mann–Whitney test using ranks with and without the motif clearly showed that the ATTG motif (8-mer) is the binding motif of the OsWOX13 TF. Similarly, the oligomer occurrence and point mutations at distinct positions were also analyzed using 5-, 6-, 7-, 8-, and 10-mers (Fig. S4).

These data showed that ATTGATTG might be a motif of OsWOX13. An extended motif was constructed using ATTGATTG as a template by adding a base in either the 5′ or 3′ direction (Fig. 3c). For example, GATTGATTG (− 1) was chosen from analysis of the 8-mer, which was extended in the 5′ direction with the base G to make GATTGATTG, and repeated analysis showed that T is the farthest in the 5′ direction (− 2). Similarly, G and T were added in the 3′ positions of + 1 and + 2, respectively, which produced TGATTGATTGGT. These data were confirmed by counting the actual occurrence of nt flanking ATTGATTG. A total of 3243 genes in rice contained the ATTGATTG motif in the 1-kb promoter regions, and 29,379 genes were retrieved from RAP-DB (http://rapdb.dna.affrc.go.jp/). The preferred nucleotides were searched (Fig. S5) for in-flanking sequences around ATTGATTG. A and T were preferable at − 3 and − 2, and G and A were preferable at the − 1 position. By contrast, A/G was preferable at the + 1 position, and T was preferable at the + 2 and + 3 positions.

Among 36,790 feature probes, the 646 probes contained the ATTGATTG motif (Fig. 2a, Table S4). From these probes, we identified 635 putative feature genes of *OsWOX13*. Gene ontology (GO)-based functional enrichment analysis of the above candidate genes was performed using the web-based tool AgriGO (http://bioinfo.cau.edu.cn/agriGO/analysis.php). The results revealed that among the 635 genes, 501 were annotated, among which 10 GO terms showed significant differences compared with those in the *Oryza sativa* database as a background reference (Table 2). The most enriched terms of macromolecule metabolic process (GO:0043170) were significantly enriched, including protein (GO:0019538), carbohydrate (GO:0005975), lipid (GO:0006629), and nucleobase (GO:0006139) (Table 2).

**Table 2:** Statistical analysis of the putative feature genes of OsSMF1 and OsWOX13 by AgriGO

Transcription factor	ID	GO Term	Query item	*p* value
OsWOX13^a^	GO:0043170	Macromolecule metabolic process	92	1.30E-46
	GO:0019538	Protein metabolic process	48	2.60E-27
	GO:0006950	Response to stress	21	2.80E-18
	GO:0006139	Nucleobase, nucleoside, nucleotide and nucleic acid metabolic process	41	9.30E-17
	GO:0005975	Carbohydrate metabolic process	20	6.10E-15
	GO:0045449	Regulation of transcription	27	2.60E-14
	GO:0051171	Regulation of nitrogen compound metabolic process	27	3.00E-14
	GO:0006629	Lipid metabolic process	9	0.0000014
	GO:0016265	Death	7	0.0011
	GO:0007154	Cell communication	6	0.0024
OsSMF1^b^	GO:0043170	Macromolecule metabolic process	154	2.10E-88
	GO:0019538	Protein metabolic process	75	5.40E-45
	GO:0006139	Nucleobase, nucleoside, nucleotide and nucleic acid metabolic process	79	2.70E-40
	GO:0045449	Regulation of transcription	41	3.00E-22
	GO:0051171	Regulation of nitrogen compound metabolic process	41	3.70E-22
	GO:0051276	Chromosome organization	18	3.90E-21
	GO:0006950	Response to stress	17	1.40E-11
	GO:0005975	Carbohydrate metabolic process	19	1.50E-11
	GO:0006629	Lipid metabolic process	15	5.30E-11
	GO:0034660	ncRNA metabolic process	6	1.3E-07
	GO:0016051	Carbohydrate biosynthetic process	6	0.00002

The 635 putative feature genes of OsWOX13 (a) with the ATTGATTG motif and 932 putative feature genes of OsSMF1 (b) with the GCCACGTCA motif were chosen and subjected to gene ontology analysis using AgriGO (http://bioinfo.cau.edu.cn/agriGO/analysis.php)

Categories such as death (GO:0016265) and response to stress (GO:0006950) were also highly enriched. These results agreed with the observation in a previous paper that, compared with control plants, rice plants overexpressing *OsWOX13* showed early flowering and drought tolerance (Minh-Thu et al. 2018).

To verify putative features of OsWOX13, we selected *Hd1-3* (Os08g0536300), for which a probe (Os08g0536300_14, AATATAACGAAACATGCAATCAATCAAAATGTTGGGAAGG) contains the CAATCAAT, a ATTG motif in a reverse complementary manner (Fig. 3d and Table S1). We assayed its binding specificity to recombinant OsWOX13 by EMSA using carboxyfluorescein (FAM)-labeled double-stranded oligonucleotide probes. The binding of OsWOX13 to the 40-bp probe with the ATTG motif was detected as lagging bands (Fig. 3d). These results confirmed the ATTG motif previously identified using Q9-PBM analysis (Minh-Thu et al. 2018).

### Identifying the DNA-binding motif and putative feature genes of OsSMF1 by RPBM

OsSMF1 reportedly binds multiple cis-elements (Kim et al. 2017). To verify this finding, RPBM was applied to identify the binding motif of OsSMF1, and 32 9-mers were extracted from a 40-bp probe similar to that for OsWOX13. The 63,811 probes produced 251,910 distinct oligomers, and the total occurrence was 2,041,952 (Fig. 2b and Table S2). The average intensity and occurrence of 9-mers were 21,725.2 ± 11,270.6 and 2.75, respectively. In contrast to OsWOX13, several groups were identified by initial cluster analysis, suggesting that OsSMF1 binds several motifs. Thus, the distinct 9-mers with frequencies four times the average occurrence (over 11) were sorted according to the value of the intensity multiplied by the occurrence, and then the 9-mers were narrowed down to 648 in total (Table 3, Table S5). This list produced 4 clusters, GCCACGTCA, ACGTAAGCG, GCTGACTCA, and AGGATGCCA, with 335, 24, 31 and 24 9-mers, respectively (Table S6, Fig. 3e). In addition, these results showed that the cluster of GCCACGTCA was predominant and that other clusters were minor but distinct. In a previous paper, Q9-PBM and EMSAs were used to show that OsSMF1 binds the GCN4 (TGA(G/C)TCA), ACGT (CCACGT(C/G)), and ATGA (GGATGAC) motifs with three different affinities (Kim et al. 2017). GCCACGTCA and ACGTAAGCG are part of the ACGT motif, GCTGACTCA is included in the GCN4 motif, and AGGATGCCA is very similar to the ATGA motif.

**Table 3:** 9-Mers highly ranked by intensity and containing the GCCACGTCA sequence

Rank	9-Mer	Intensity_ave	Occurrence_total	Int_ave*Occur_tot	Occur_diff_pos
1	GCCACGTCA	24,766.52	834	20,655,280	31
2	TGACGTGGC	23,386.91	326	7,624,133	31
3	CCACGTCAG	24,362.08	533	12,984,991	31
4	TGCCACGTC	23,036.39	481	11,080,503	31
5	CACGTCAGC	24,206.7	448	10,844,602	29
6	CGCCACGTC	23,606.49	452	10,670,132	31
7	GCGCCACGT	22,976.78	341	7,835,082	31
8	GCCACGTGG	20,062.16	332	6,660,638	31
9	CCACGTGGC	19,612.18	338	6,628,917	31
10	ATGCCACGT	22,132.63	296	6,551,258	31
11	CTGCCACGT	21,409.31	287	6,144,472	31
12	CCACGTCAT	23,338.93	241	5,624,681	30
13	TGCCACGTA	23,632.13	235	5,553,550	30
14	CCACGTCAC	22,655.01	243	5,505,168	30
15	GCCACGTAG	21,171.72	239	5,060,042	31
16	TGCCACGTG	20,892.83	241	5,035,173	31
17	TTGCCACGT	24,255.62	207	5,020,914	30
18	GTGCCACGT	21,387.37	224	4,790,770	30
19	CTGACGTGG	23,467.43	199	4,670,019	31
20	TCCACGTCA	21,404.54	216	4,623,380	31
55	GCTGACTCA	17,418.66	144	2,508,287	31
56	TGACTCAGC	17,403.51	144	2,506,105	29
67	CTGACTCAG	18,380.93	121	2,224,092	30
82	GGATGCCAC	24,137.48	81	1,955,136	26
103	GCTGAGTCA	16,726.02	99	1,655,876	27
105	AGGATGCCA	23,742.69	68	1,614,503	26

The column descriptions are the same as those for Table 1

Since the GCCACGTCA and ACGTAAGCG clusters have ACGT motifs, they were aligned together and provided a position matrix, and CCACGTCA was a main element (Fig. 3f). The distribution of the CCACGTCA motif among the RPBM features was also depicted (Fig. S2 b). The number of features with the forward motif, CCACGTCA, was 1561 with a mean intensity of 20,142.0 ± 12,450.6, and 85 were found in the tail regions. The number of features with the reverse complementary motif, TGACGTGG, was 723, and the mean intensity was 17,400.6 ± 10,715.1; 62 features were found in the tail region. Among 2431 with the element, 2284 (94.0%) were found in the strong binding zone. The number of features without the motif (None_cis_features) was 938,519, and the mean intensity was 1931.3 ± 3125.6. Thus, the signal intensities of the features containing the forward and reverse complementary motifs were 10.4- and 9.0-fold higher, respectively, than those of the features without the motif. The Wilcoxon–Mann–Whitney test was performed as shown for the feature probes containing CCACGTCA and those without the sequence, and it produced a *p* value of 0, suggesting that CCACGTCA contributed significantly to binding. To test the preferences for any nucleotide flanking CCACGTCA sequences, an extended motif was constructed using CCACGTCA as a template by adding a base in either the 5′ or 3′ direction with OsWOX13 (Fig. 3g). Mutation analysis was performed with OsWOX13 by changing the bases in each CCACGTCA (Fig. 3g). The intensities strongly decreased with changes to A at the 3rd position (by 10,637.3) and A at the 7th position (by 8356.0). An extended motif was constructed using CCACGTCAG as a template by adding a base in either the 5′ or 3′ direction, producing TGCCACGTCAGC. Thus, this study showed that CCACGTCA is a DNA-binding motif for OsSMF1, while the flanking sequences of this motif showed no significant effect. Similarly, the intensities of the feature probes in terms of the occurrence and mutations at each position were also analyzed with 5-, 6-, 7-, 8-, 10-, and 11-mers (Fig. S6). The feature probes containing CCACGTCA (932) are listed in Table S7.

Among 63,811 probes with an intensity of 3137, 932 probes contained the CCACGTCA sequence, from which 890 putative feature genes were identified for *OsSMF1* (Fig. 2, Table S8). When 687 genes among these candidate genes were subjected to GO analysis using AgriGO, “macromolecule metabolic process” was also highly abundant, similar to GO analysis of OsWOX13 (Table 2). Several GO terms were enriched, such as “carbohydrate biosynthetic process (GO:0016051)”, “regulation of nitrogen compound metabolic process (GO:0051171)”, “ncRNA metabolic process (GO:0034660)”, and “chromosome organization (GO:0051276)” (Table 2).

To verify putative features of OsSMF1, we selected two nonapical meristem (NAM) proteins, Os01g0393100 (ONAC026) and Os05g0415400 (ONAC024), from “regulation of nitrogen compound metabolic process (GO:0051171). ONAC026 and ONAC024 were identified as feature genes of OsSMF1 in a previous paper (Kim et al. 2017). Probes from the ONAC026 and ONAC024 promoters contained the ACGT and GNC4 motifs, respectively (Fig. 3h). We assayed their binding specificities to recombinant OsSMF1 by EMSA using FAM-labeled double-stranded oligonucleotides corresponding to each probe. The binding of OsSMF1 to the 40-bp probes was detected as lagging bands (Fig. 3h). This result indicated that OsSMF1 directly binds to the promoters of ONAC026 and ONAC024. These results indicate that OsSMF1 has multiple distinct motifs, with OsSMF1 binding to the ACGT (CCACGT(C/G)), GCN4 (TGA(G/C)TCA), and ATGA (GGATGAC) motifs.

### Identifying the DNA-binding motif of OsWRKY34 by RPBM

WRKY TFs are encoded by one of the largest families in plants and are involved in biotic and abiotic stress responses as well as development processes (Wu et al. 2005). RPBM was also applied to identify the new binding motif of OsWRKY34. When the overexpression vector for OsWRKY34 was introduced into a wild-type plant, it showed reverse-folded leaf phenotypes (manuscript in preparation). The distribution of the RPBM features was also depicted. Experiments with OsWRKY34 also provided a similar rank-ordered signal distribution, showing a steep slope on the left followed by a heavy right tail (Fig. S7a). The number of features with an intensity higher than background was 954,573, and the mean intensity was 5330.2 ± 2535.6. (Fig. S7a). Regarding OsWRKY34 analysis, 67,864 probes with an intensity higher than 8023 were selected (Table S8) as described above. RPBM was applied to identify the binding motif of OsWRKY34, and 32 9-mers were extracted from a 40-bp probe in the same manner as for OsWOX13. The 67,864 probes provided 241,284 distinct oligomers, and the total occurrence was 2,17,640 (Fig. S8). The average intensity and occurrence of 9-mers were 9863.2 ± 2941.4 and 2.0, respectively. The average intensities were slightly bit lower than those of OsWOX13 and OsSMF1.

**Fig. 4:**
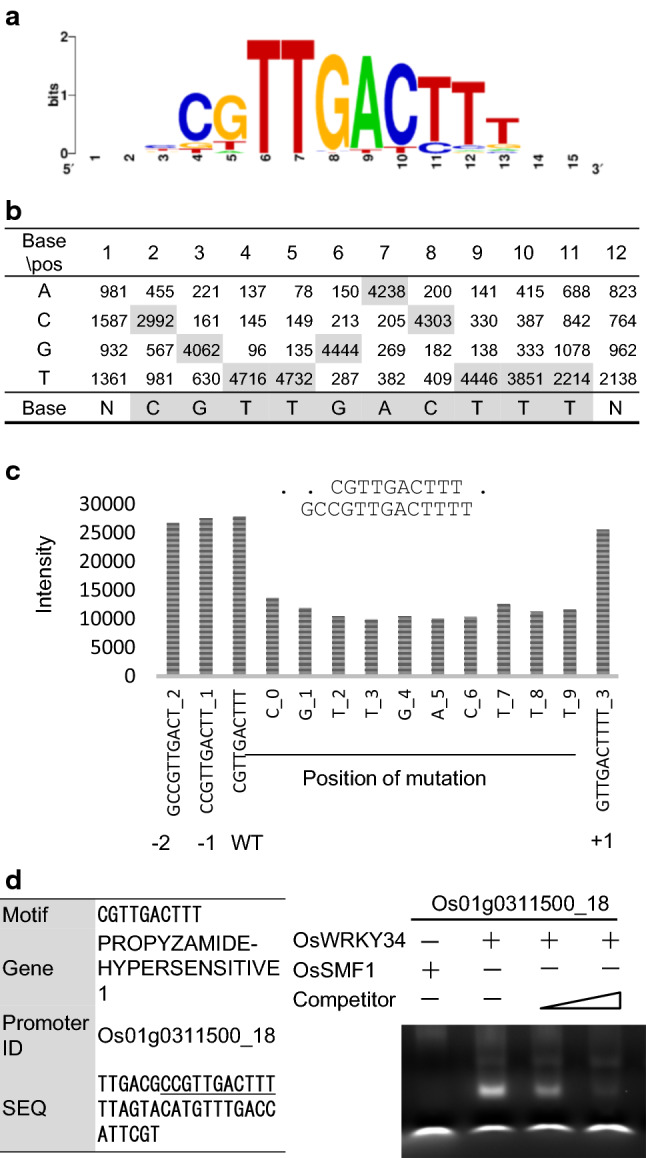
DNA-binding motif analysis of OsWRKY34. **a** DNA-binding motifs of OsWRKY34 determined by clustering of the significant binding sequences. They were visualized using the Web logo program (weblogo.berkeley.edu). **b** Position weight matrix from the clustering of 10-mers. **c** Comparison of the intensities of oligomers with point mutations at distinct positions in CGTTGACTTT. Binding motif of OsWRKY34 from the Wilcoxon–Mann–Whitney test, *p* value 0. **d** EMSA-based competition analysis of OsWRKY34 using the probe Os01g0311500_18, which contains the CGTTGACTTT motif. The 40-bp sequences used as probes and their competitors are depicted. EMSAs were performed using the OsWRKY34:DsRed protein and a probe 5′-labeled with FAM. Competition for the labeled sequences was tested by adding different concentrations of unlabeled probe

These 9-mers were sorted according to their intensities, and CGTTGACTT had the highest intensity of 25,164 with an occurrence of 361 (Table 4). To identify a consensus sequence, cluster analysis was performed such that any 9-mer with a 5-nt sequence matching the template of the highest intensity belonged to a group. The 2021 9-mers formed a cluster with CGTTGACTT as a template. These top 20 9-mers were ranked by intensity (Table 4). Web logo (weblogo.berkeley.edu) provided the 10-mer CGTTGACTTT, which was a base longer than the 9-mer CGTTGACTT that was initially identified (Fig. 4a).

**Table 4:** 9-Mers highly ranked by intensity and containing the CGTTGACTT sequence

Rank	Nine-mer	Intensity_ave	Occurrence_total	Int_Ave*Occur_tot	Occur_diff_pos
1	CGTTGACTT	25,164.44	361	9,084,364	31
2	CCGTTGACT	24,951.13	315	7,859,606	31
3	GCCGTTGAC	23,632.71	212	5,010,135	31
4	GTTGACTTT	23,401.82	436	10,203,193	31
5	CACCGTTGA	23,370.05	113	2,640,816	31
6	ACGCCGTTG	23,263.98	156	3,629,181	30
7	TGACGCCGT	23,085.01	136	3,139,562	29
8	GACGCCGTT	23,041.92	142	3,271,953	30
9	GACTTTTTA	22,948.16	222	5,094,491	31
10	ACCGTTGAC	22,852.19	144	3,290,716	31
11	GACACCGTT	22,722.73	85	1,931,432	29
12	TGACACCGT	22,623.96	81	1,832,541	27
13	CGCCGTTGA	22,514.94	188	4,232,808	31
14	ACACCGTTG	22,474.54	105	2,359,827	30
15	TTGACGCCG	22,404.72	72	1,613,140	26
16	ATGACGCCG	22,054.86	63	1,389,456	24
17	ACTTTTTAG	21,757.46	114	2,480,350	30
18	TATGACGCC	21,719.33	39	847,054	22
19	GTATGACGC	21,634.89	37	800,491	18
20	TTGACACCG	21,561.64	73	1,574,000	25

The column descriptions are the same as those for Table 1

The distribution of the CGTTGACTTT motif among the RPBM features was also depicted (Fig. S7 b). The number of features with the forward motif CGTTGACTTT was 304, with a mean intensity of 27,855.3 ± 10,062.0; 1 was found in the tail regions. The reverse complementary motif AAAGTCAACG numbered 231, and its mean intensity was 12,158.2 ± 3011.1; 119 features were found in the tail region. In total, 655 features had the element, 535 (81.7%) of which were found in the strong binding zone. The number of features without the motif was 953,918, with a mean intensity of 5321.2 ± 2495.2. Thus, the signal intensities of the features containing the forward and reverse complementary motifs were 5.2- and 2.3-fold higher, respectively, than those of the features without the motif. In addition, the feature intensity of the forward motif was 2.3-fold higher than that of the reverse.

The presence of nucleotides at each position is shown in a PWM by the clustering of these 10-mers (Fig. 4b). In addition, mutation analysis was conducted by changing bases in each CGTTGACTTT (Fig. 4c). A base-mutated sequence provided a maximum decrease at the 4th nt, T, and a minimum at the 1st nt, C (9768 and 12,279.6, respectively). Similarly, oligomer occurrence and point mutations at distinct positions were also analyzed using 5-, 6-, 7-, 8-, and 9-mers (Fig. S9).

Among the 67,864 feature probes from the steep region, 301 contained the CGTTGACTTT motif (Fig. S8, Table S9). From these probes, we identified 195 putative feature genes of *OsWRKY34*. GO-based functional enrichment analysis of the above candidate genes was performed using the web-based tool AgriGO (http://bioinfo.cau.edu.cn/agriGO/analysis.php). Among the 195 genes, 162 were annotated, of which 9 GO terms showed significant differences compared with those in the *Oryza sativa* database as a background reference. Categories such as electron carrier activity (GO:0009055) and membrane-bounded vesicle (GO:0031988) were highly enriched.

To verify the putative features of OsWRKY34, we selected PROPYZAMIDE-HYPERSENSITIVE 1 (Os01g0311500), for which a probe (Os01g0311500 _18,

TTGACGCCGTTGACTTTTTAGTACATGTTTGACCATTCGT) contained the CGTTGACTTT sequence (Fig. 4 and Table S9). We assayed its binding specificity to recombinant OsWRKY34 by EMSA using carboxyfluorescein (FAM)-labeled double-stranded oligonucleotide probes. The binding of OsWRKY34 to the 40-bp probe was detected as lagging bands (Fig. 4d).

## Discussion

To exploit the cis-elements around promoter regions, we designed an RPBM where 1 kb of the promoter and 5′ untranslated region was covered by overlapping 40-bp feature probes. The initial signal distribution of RPBM was very similar to that of Q9-PBM, where quadruple 9-mer oligonucleotides were designed as the feature probes (Kim et al. 2009). These results suggest that the binding of TFs and their cognate binding sites in RPBM are as stable as those found in Q9-PBM. The probe design from the promoter regions overcomes potential complexities because of the concatemers of feature sites, and the binding in the promoter regions is understood. The analysis of signal intensities of 5–10 oligomers, particularly 9-mers, highlighted putative binding sequences, and the comparison of those signals of oligomers with point mutation at each site clearly showed strong binding sequences. These findings further confirmed that the feature probes on RPBM can be directly used in subsequent EMSA analysis without additional modification.

We first applied 9-mer-based analysis and identified the ATTGATTG DNA-binding sequence and 635 putative feature genes of *OsWOX13*,* which has* one dominant binding site. The Plant Transcription Factor Database (Jin et al. 2017; planttfdb.gao-lab.org/) showed that Os01g0818400 (OsWOX8) has a representative motif, CAATCAA, which has a 7-nt sequence of the reverse complement of ATTGATTG. Many homeobox-containing TFs contain ATTGATTG or parts of it in their motifs, and this is also found in similar homeobox TFs, as shown in Os090528200 and Os03g0170600 in PlantTFDB. We also surveyed the UniPROBE database (Hume et al. 2015) and compared its entries with putative cis-elements of homeo-domain-containing TFs such as UP00615B_1 and UP00158A_1 from humans and mice, respectively. These factors also provided various GA- or AT-rich motifs. In particular, the UP00158A_1-binding site contains AATTAATTA and ATTA repeats and showed a base (A to G) difference with ATTG repeats in the ATTGATTG motif in our analysis (Minh-Thu et al. 2018).

The mode by which OsSMF1 modulates downstream TFs bound to GCCACGTCA and ACGTAAGCG, which include the ACGT motif, might be complex. GCTGACTCA is included in the GCN4 motif GGATGCC, which is very similar to the ATGA motif, and the cluster near CCACGTCA is predominant, confirming previous results (Kim et al. 2017). Although the cis-elements are not registered in PlantTFDB, they represent the basic leucine zipper in the database, consistent with those found in many basic leucine zipper TFs. These TFs contain an ACGT motif in their representative binding motif. A few examples are Os01g0859500 with GATGACGTCA, Os02g0203000 with TGATGACGTGGC, Os02g0766700 with TGCCACGTGNCC, and Os03g0796900 with TGACGTGG, which are reverse complementary to CCACGTCA (Table S10). These results suggest that OsSMF1 evolved to have specific functionality involving common DNA-binding activity due to the bZIP domain.

Since the analysis of RPBM with OsWOX13 and OsSMF1 confirmed the results of these TFs previously found in Q9-PBM, we adopted the technology to identify unknown cis-elements of OsWRKY34. Initially, 9-mers provided the highest intensity of CGTTGACTT. However, best alignments and mutation analysis with other 9-mers provided a putative 10-mer element, CGTTGACTTT. The elements might be extended to even CGTTGACTTTTT. Thus, among 301 feature probes on RPBM, 239 had these elements. In addition, many of these elements were followed by another DNA sequence motif (T)(T)TGAC(C/T), known as the W box (Eulgem et al. 2000). OsWRKY71, a transcriptional repressor of GA signaling in aleurone cells, binds specifically to TGAC-containing W boxes of the *Amy32b* promoter (Zhang et al. 2004). In this paper, we found that the DNA-binding motif of OsWRKY34 was CGTTGACTTT. Of 301 probes containing the DNA sequence motif CGTTGACTTT, the 99 probes contained two W boxes with TGAC as the core motif. Thus, CGTTGACTTT might comprise W boxes and might be involved in the regulation of subsequent gene expression. In our preliminary data, OsWRKY34 overexpression in the wild-type plants showed a reverse-folded leaf phenotype, and the involved genes are being investigated.

Application of the technology might even be expanded for TFs that are heterodimers or form higher-order complexes, because a 40-nt probe could have additional putative cis-elements. Although cis-elements in a promoter are spaced in many cases, some, such as auxin response elements, are enriched in narrow regions with bZIP response elements and Myb response elements (Berendzen et al. 2012). In addition, extended analysis of the databases can be evaluated with other interacting TFs that may be functionally associated in processes such as metabolism and development. For example, the TFs that might be associated with OsWOX13 were sought in PlantTFDB through elements in the 40 bp flanking ATTGATTG in the promoter regions (data not shown). Thus, the CAATCA site for Os09g0528200 (homeobox-leucine zipper protein), the AAAAAG site for Os02g0707200 (Dof-like protein 34) and the CAAGNAA site for Os03g0119966 (NAC-domain protein) are the frequently found elements in rice.

## Conclusions

The present study showed that RPBM can be applied to analyze DNA-binding motifs with a TF where binding is evaluated in extended natural promoter regions. The analysis can also be applied to TFs with single or multiple binding motifs. The technology may even be expanded for application to TFs that are heterodimers or form higher-order complexes. In addition, extended analysis of the databases can be evaluated with other interacting TFs that may be functionally associated in processes such as metabolism and development.

### Author contribution statement

JSK generated the data and wrote the paper. SC and KMJ performed the flanking DNA sequencing analysis. GSL observed the field phenotypes of the rice lines. KK and JSJ analyzed the binding motifs in the databases. YKK inspired the overall work and revised the final manuscript. All the authors read and approved the final manuscript.

## Supplementary Information

Below is the link to the electronic supplementary material.

Supplementary file1 (PPTX 230 KB)

Supplementary file2 (XLSX 9693 KB)

## Abbreviations

PBM: Protein-binding microarray
TF: Transcription factor
RPBM: Rice (*Oryza sativa*)-specific protein-binding microarray
ChIP: Chromatin immunoprecipitation
PCR: Polymerase chain reaction
