# Supplementary material for: Rice protein-binding microarrays: a tool to detect cis-acting elements near promoter regions in rice
Source: Planta. 2021 Jan 21;253(2):40. doi: 10.1007/s00425-021-03572-w (PMC7819943; doi:10.1007/s00425-021-03572-w)
Supplement: Supplementary file 1 — Supplementary file1 (PPTX 230 KB) [file 425_2021_3572_MOESM1_ESM.pptx]

## Slide 1
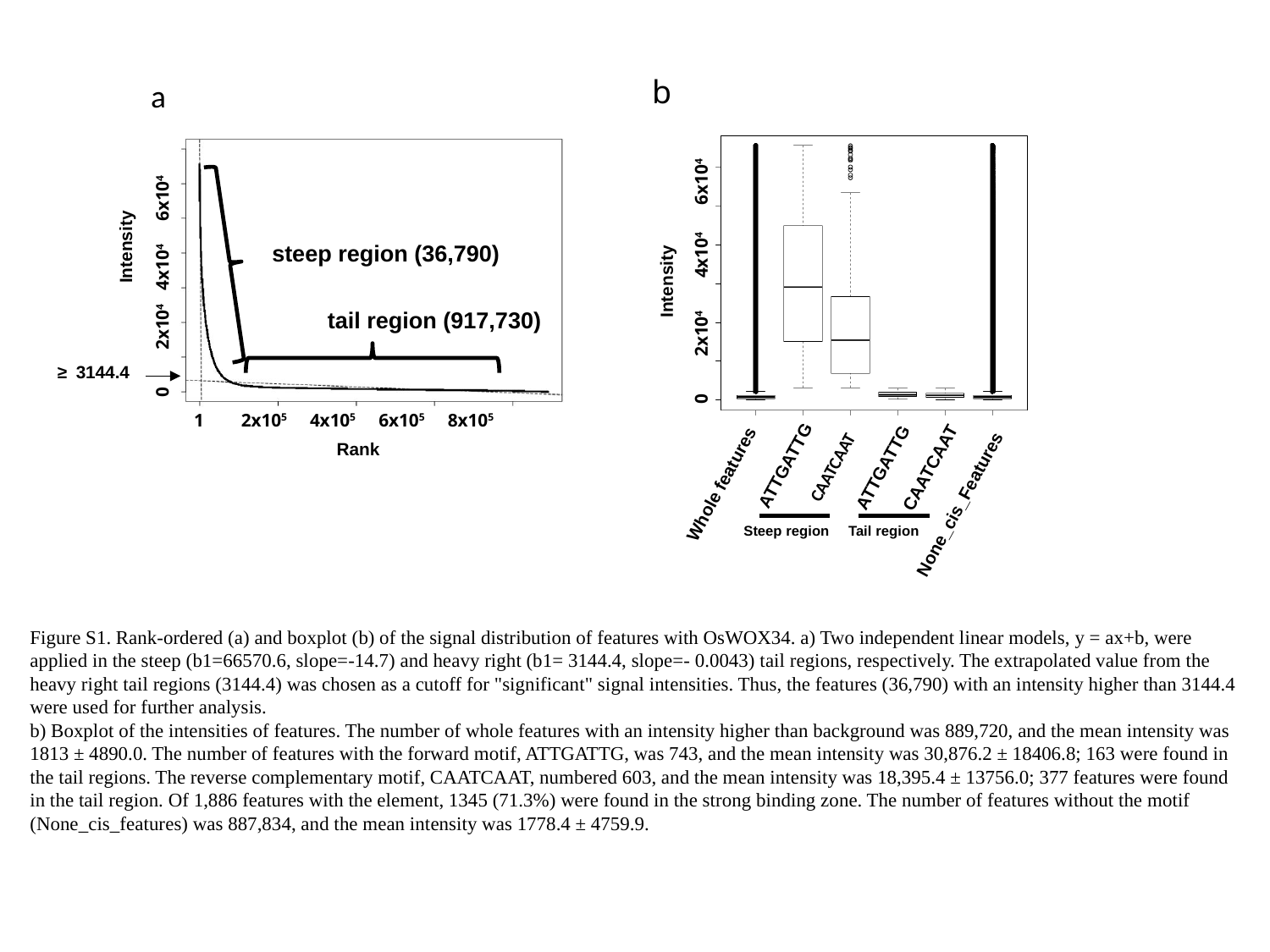

b
a
0 2x10^4^ 4x10^4^ 6x10^4^
Intensity
steep region (36,790)
0 2x10^4^ 4x10^4^ 6x10^4^
Intensity
tail region (917,730)
≥ 3144.4
1 2x10^5^ 4x10^5^ 6x10^5^ 8x10^5^
Rank
CAATCAAT
CAATCAAT
ATTGATTG
ATTGATTG
Whole features
None_cis_Features
Tail region
Steep region
Figure S1. Rank-ordered (a) and boxplot (b) of the signal distribution of features with OsWOX34. a) Two independent linear models, y = ax+b, were applied in the steep (b1=66570.6, slope=-14.7) and heavy right (b1= 3144.4, slope=- 0.0043) tail regions, respectively. The extrapolated value from the heavy right tail regions (3144.4) was chosen as a cutoff for "significant" signal intensities. Thus, the features (36,790) with an intensity higher than 3144.4 were used for further analysis.
b) Boxplot of the intensities of features. The number of whole features with an intensity higher than background was 889,720, and the mean intensity was 1813 ± 4890.0. The number of features with the forward motif, ATTGATTG, was 743, and the mean intensity was 30,876.2 ± 18406.8; 163 were found in the tail regions. The reverse complementary motif, CAATCAAT, numbered 603, and the mean intensity was 18,395.4 ± 13756.0; 377 features were found in the tail region. Of 1,886 features with the element, 1345 (71.3%) were found in the strong binding zone. The number of features without the motif (None_cis_features) was 887,834, and the mean intensity was 1778.4 ± 4759.9.

## Slide 2
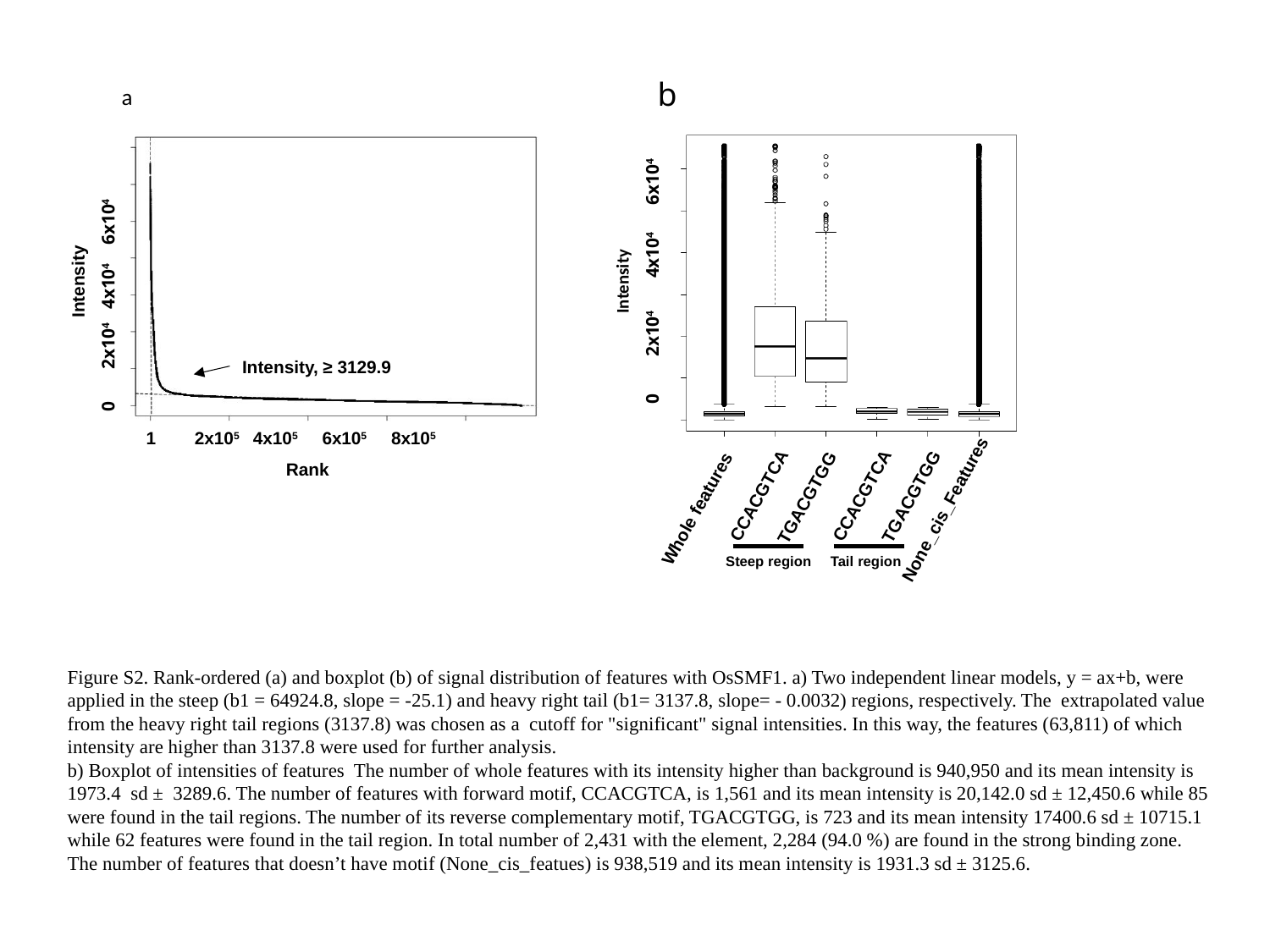

b
a
0 2x10^4^ 4x10^4^ 6x10^4^
0 2x10^4^ 4x10^4^ 6x10^4^
Intensity
Intensity
Intensity, ≥ 3129.9
1 2x10^5^ 4x10^5^ 6x10^5^ 8x10^5^
Rank
CCACGTCA
CCACGTCA
TGACGTGG
TGACGTGG
None_cis_Features
Whole features
Tail region
Steep region
Figure S2. Rank-ordered (a) and boxplot (b) of signal distribution of features with OsSMF1. a) Two independent linear models, y = ax+b, were applied in the steep (b1 = 64924.8, slope = -25.1) and heavy right tail (b1= 3137.8, slope= - 0.0032) regions, respectively. The extrapolated value from the heavy right tail regions (3137.8) was chosen as a cutoff for "significant" signal intensities. In this way, the features (63,811) of which intensity are higher than 3137.8 were used for further analysis.
b) Boxplot of intensities of features The number of whole features with its intensity higher than background is 940,950 and its mean intensity is 1973.4 sd ± 3289.6. The number of features with forward motif, CCACGTCA, is 1,561 and its mean intensity is 20,142.0 sd ± 12,450.6 while 85 were found in the tail regions. The number of its reverse complementary motif, TGACGTGG, is 723 and its mean intensity 17400.6 sd ± 10715.1 while 62 features were found in the tail region. In total number of 2,431 with the element, 2,284 (94.0 %) are found in the strong binding zone. The number of features that doesn’t have motif (None_cis_featues) is 938,519 and its mean intensity is 1931.3 sd ± 3125.6.

## Slide 3
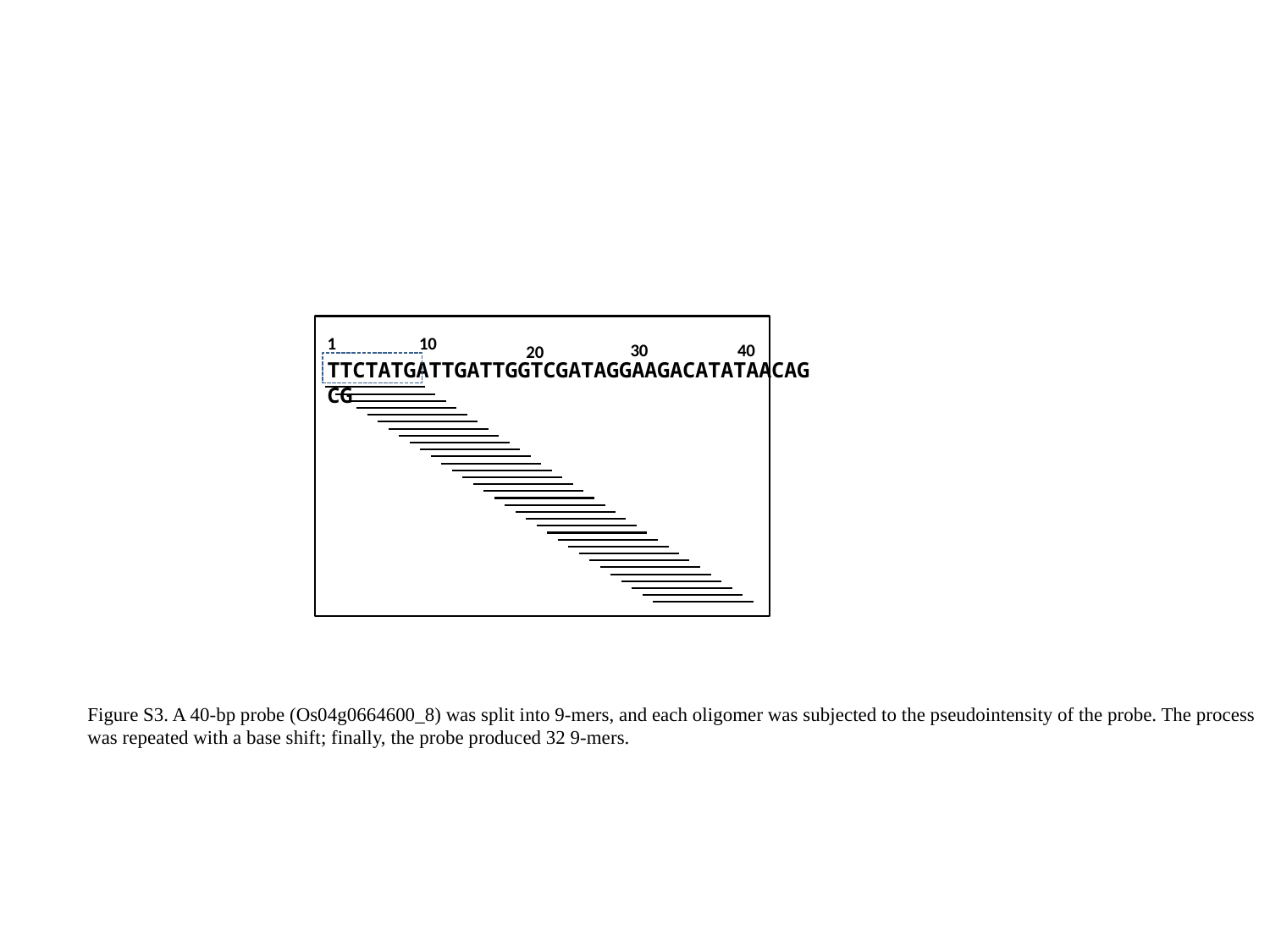

1
10
30
40
20
TTCTATGATTGATTGGTCGATAGGAAGACATATAACAGCG
Figure S3. A 40-bp probe (Os04g0664600_8) was split into 9-mers, and each oligomer was subjected to the pseudointensity of the probe. The process was repeated with a base shift; finally, the probe produced 32 9-mers.

## Slide 4
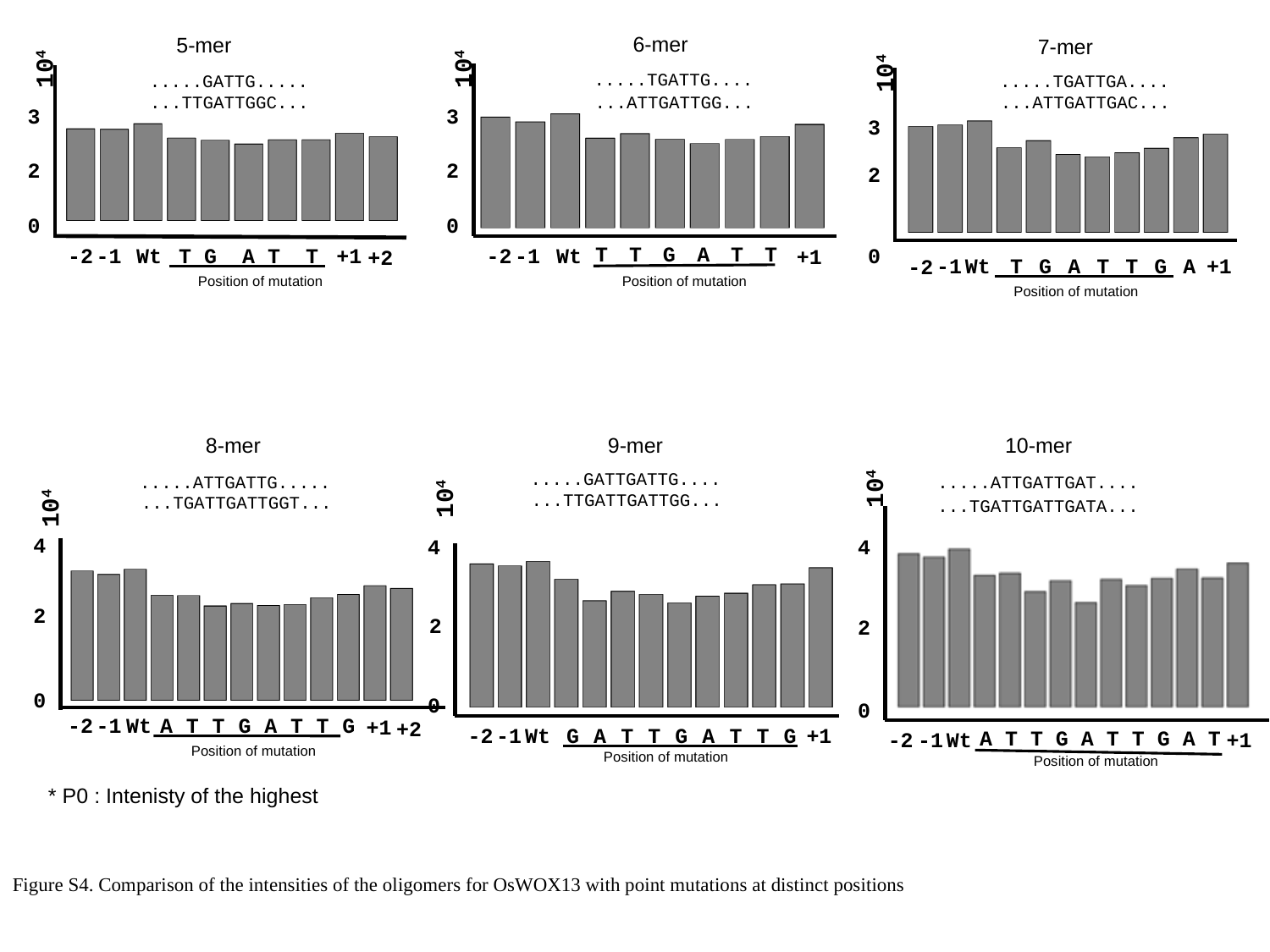

6-mer
5-mer
7-mer
104
104
104
.....TGATTG....
.....GATTG.....
.....TGATTGA....
...TTGATTGGC...
...ATTGATTGG...
...ATTGATTGAC...
3
3
3
2
2
2
0
0
T T G A T T
0
-2
-1
Wt
T G A T T
-2
-1
Wt
+1
+1
+2
-1
Wt
+1
T G A T T G A
-2
Position of mutation
Position of mutation
Position of mutation
8-mer
9-mer
10-mer
.....GATTGATTG....
.....ATTGATTGAT....
.....ATTGATTG.....
104
104
...TTGATTGATTGG...
...TGATTGATTGGT...
104
...TGATTGATTGATA...
4
4
4
2
2
2
0
0
0
-2
-1
Wt
A T T G A T T G
+1
+2
-2
-1
Wt
G A T T G A T T G
+1
A T T G A T T G A T
-2
-1
Wt
+1
Position of mutation
Position of mutation
Position of mutation
* P0 : Intenisty of the highest
Figure S4. Comparison of the intensities of the oligomers for OsWOX13 with point mutations at distinct positions

## Slide 5
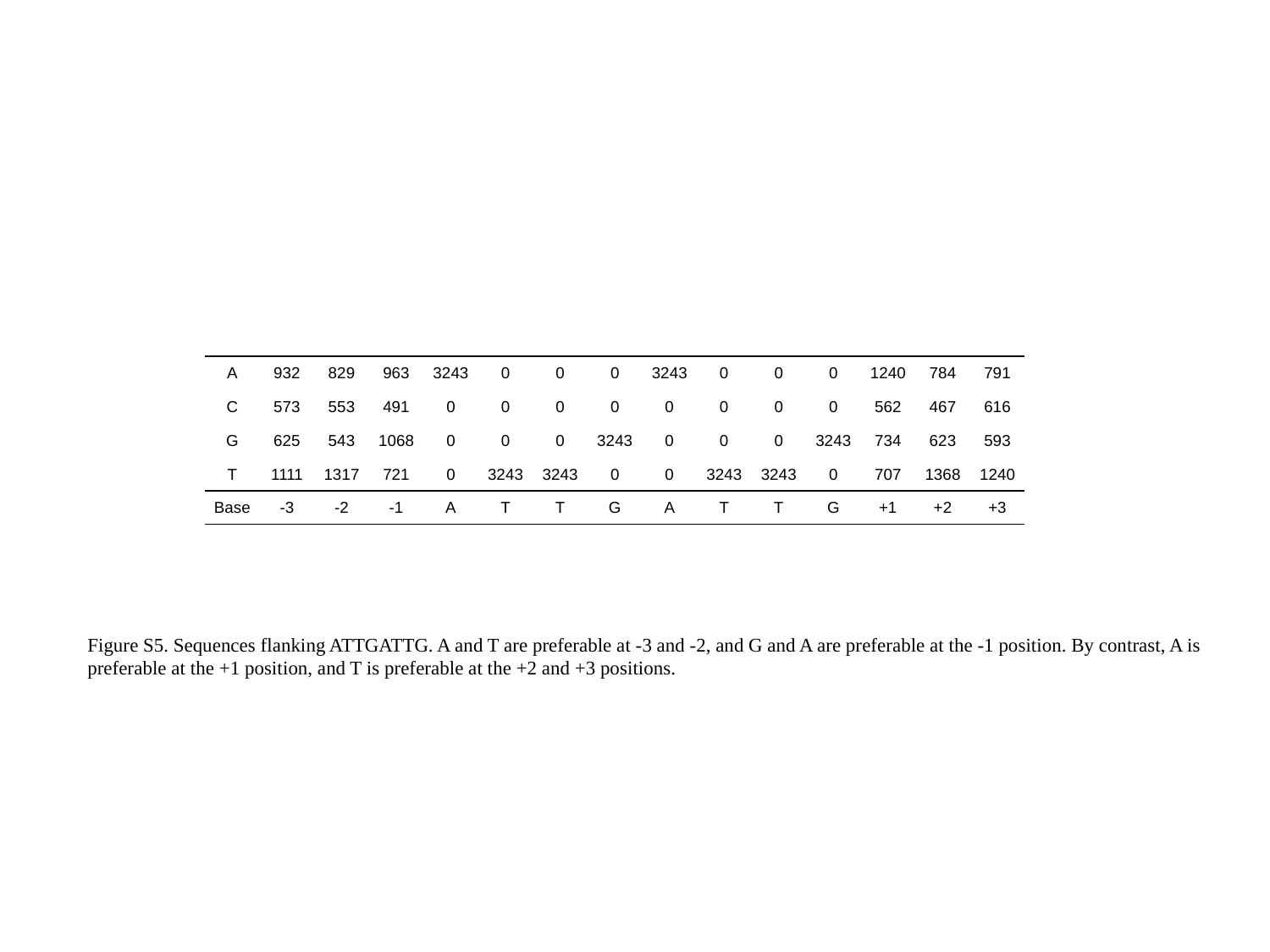

| A | 932 | 829 | 963 | 3243 | 0 | 0 | 0 | 3243 | 0 | 0 | 0 | 1240 | 784 | 791 |
| --- | --- | --- | --- | --- | --- | --- | --- | --- | --- | --- | --- | --- | --- | --- |
| C | 573 | 553 | 491 | 0 | 0 | 0 | 0 | 0 | 0 | 0 | 0 | 562 | 467 | 616 |
| G | 625 | 543 | 1068 | 0 | 0 | 0 | 3243 | 0 | 0 | 0 | 3243 | 734 | 623 | 593 |
| T | 1111 | 1317 | 721 | 0 | 3243 | 3243 | 0 | 0 | 3243 | 3243 | 0 | 707 | 1368 | 1240 |
| Base | -3 | -2 | -1 | A | T | T | G | A | T | T | G | +1 | +2 | +3 |
Figure S5. Sequences flanking ATTGATTG. A and T are preferable at -3 and -2, and G and A are preferable at the -1 position. By contrast, A is preferable at the +1 position, and T is preferable at the +2 and +3 positions.

## Slide 6
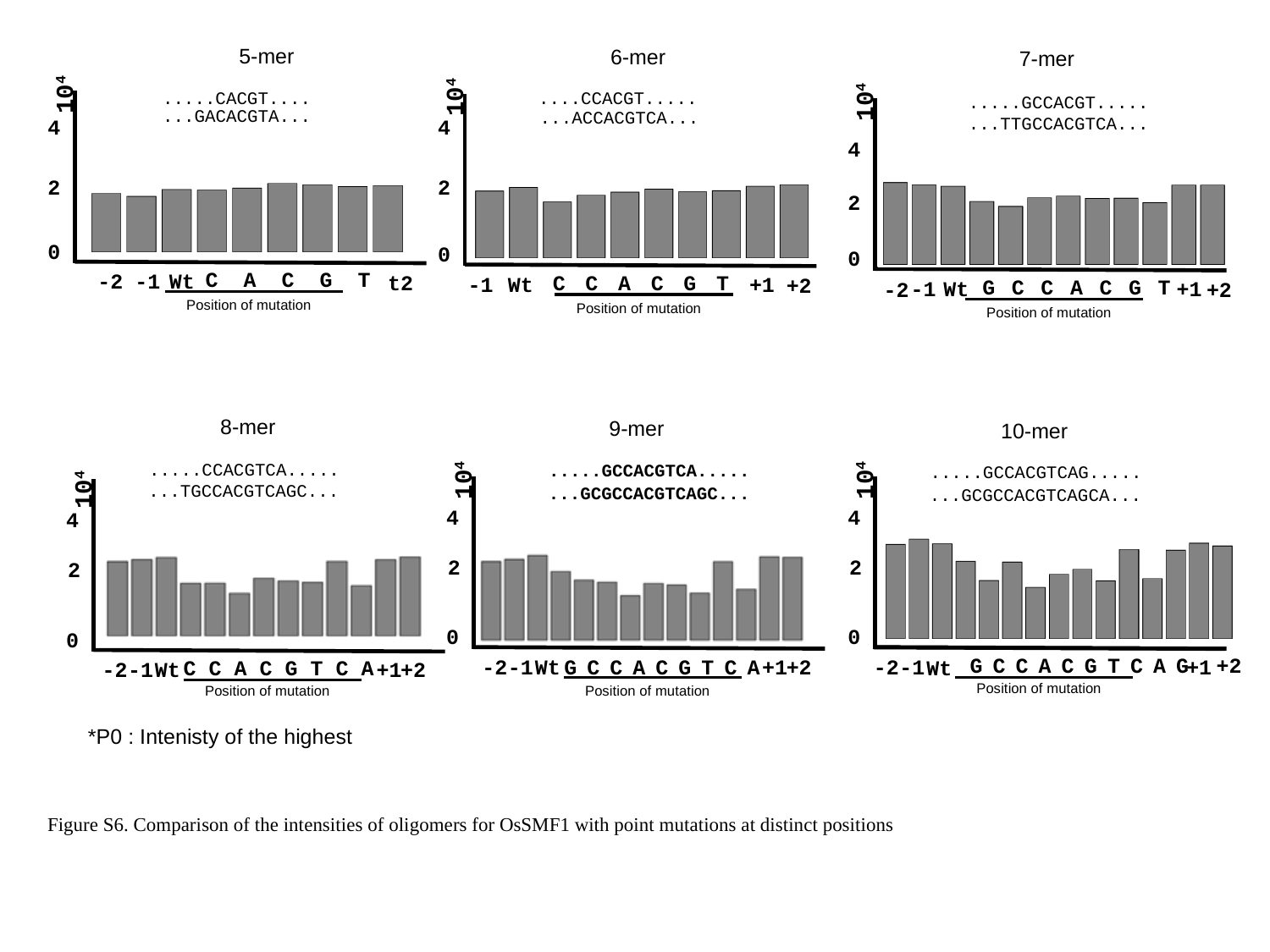

5-mer
6-mer
7-mer
104
104
....CCACGT.....
.....CACGT....
104
.....GCCACGT.....
...GACACGTA...
...ACCACGTCA...
...TTGCCACGTCA...
4
4
4
2
2
2
0
0
0
C A C G T
-2
-1
Wt
CCACGT
t2
-1
Wt
+1
+2
GCCACGT
-1
Wt
+1
-2
+2
Position of mutation
Position of mutation
Position of mutation
8-mer
9-mer
10-mer
.....CCACGTCA.....
.....GCCACGTCA.....
.....GCCACGTCAG.....
104
104
104
...TGCCACGTCAGC...
...GCGCCACGTCAGC...
...GCGCCACGTCAGCA...
4
4
4
2
2
2
0
0
0
GCCACGTCAG
+2
-2
-1
Wt
GCCACGTCA
+2
+1
+1
-2
-1
Wt
C C A C G T C A
-2
-1
Wt
+2
+1
Position of mutation
Position of mutation
Position of mutation
*P0 : Intenisty of the highest
Figure S6. Comparison of the intensities of oligomers for OsSMF1 with point mutations at distinct positions

## Slide 7
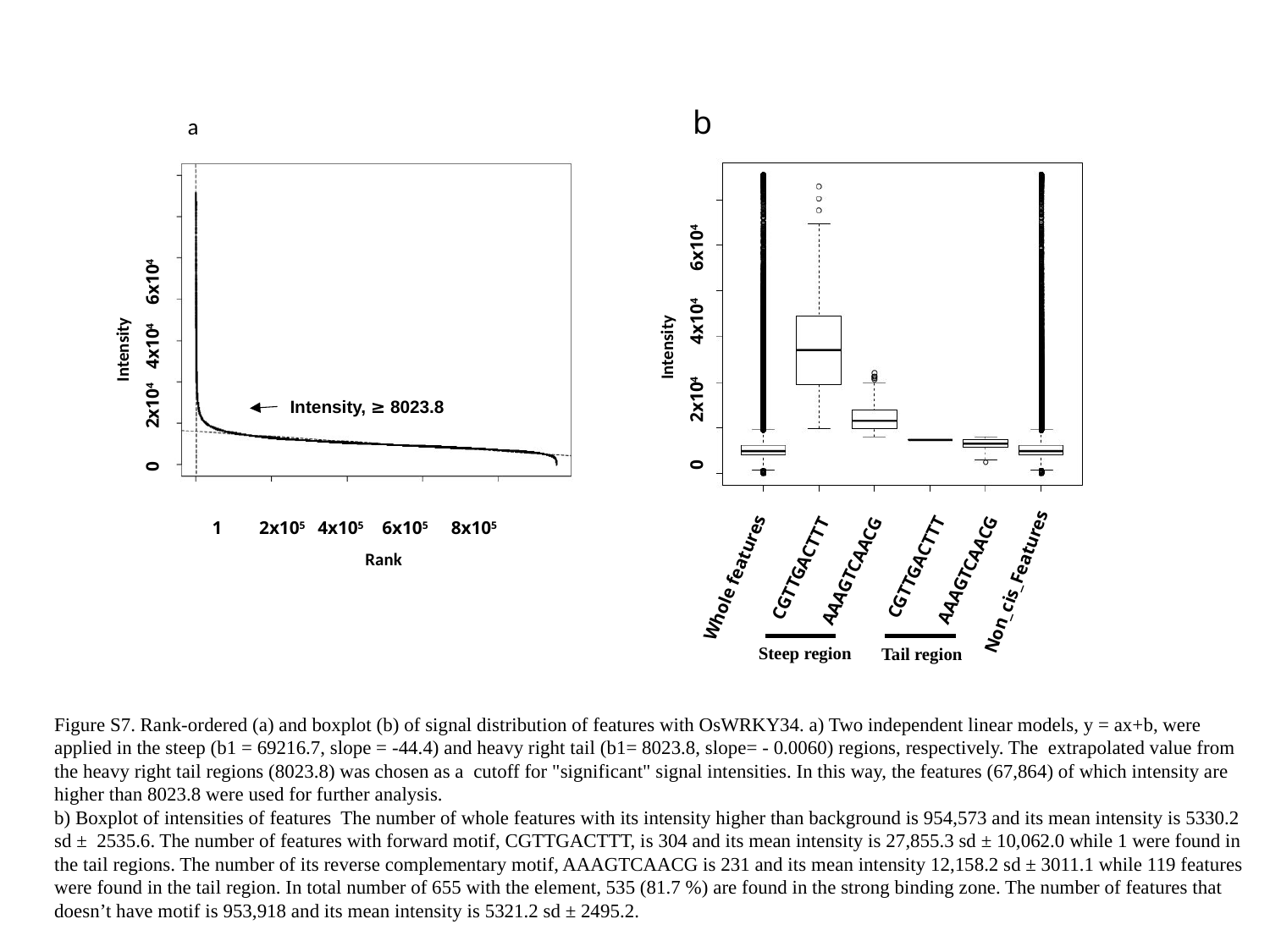

b
a
0 2x10^4^ 4x10^4^ 6x10^4^
0 2x10^4^ 4x10^4^ 6x10^4^
Intensity
Intensity
Intensity, ≥ 8023.8
1 2x10^5^ 4x10^5^ 6x10^5^ 8x10^5^
Rank
CGTTGACTTT
CGTTGACTTT
AAAGTCAACG
AAAGTCAACG
Whole features
Non_cis_Features
Steep region
Tail region
Figure S7. Rank-ordered (a) and boxplot (b) of signal distribution of features with OsWRKY34. a) Two independent linear models, y = ax+b, were applied in the steep (b1 = 69216.7, slope = -44.4) and heavy right tail (b1= 8023.8, slope= - 0.0060) regions, respectively. The extrapolated value from the heavy right tail regions (8023.8) was chosen as a cutoff for "significant" signal intensities. In this way, the features (67,864) of which intensity are higher than 8023.8 were used for further analysis.
b) Boxplot of intensities of features The number of whole features with its intensity higher than background is 954,573 and its mean intensity is 5330.2 sd ± 2535.6. The number of features with forward motif, CGTTGACTTT, is 304 and its mean intensity is 27,855.3 sd ± 10,062.0 while 1 were found in the tail regions. The number of its reverse complementary motif, AAAGTCAACG is 231 and its mean intensity 12,158.2 sd ± 3011.1 while 119 features were found in the tail region. In total number of 655 with the element, 535 (81.7 %) are found in the strong binding zone. The number of features that doesn’t have motif is 953,918 and its mean intensity is 5321.2 sd ± 2495.2.

## Slide 8
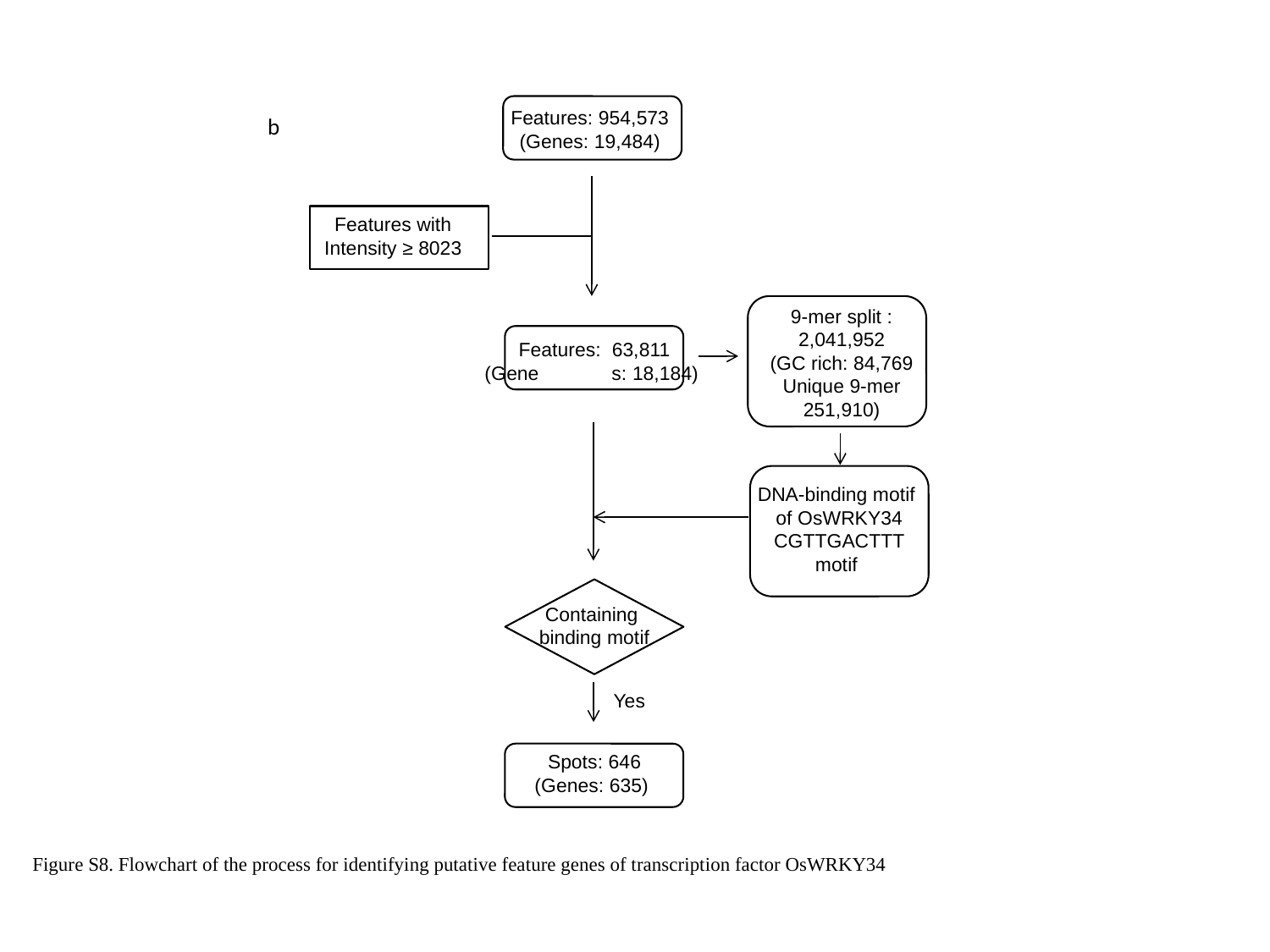

Features: 954,573
(Genes: 19,484)
b
Features with
Intensity ≥ 8023
9-mer split :
2,041,952
(GC rich: 84,769
Unique 9-mer 251,910)
Features: 63,811
(Gene	s: 18,184)
DNA-binding motif
of OsWRKY34
CGTTGACTTT motif
Containing
binding motif
Yes
Spots: 646
(Genes: 635)
Figure S8. Flowchart of the process for identifying putative feature genes of transcription factor OsWRKY34

## Slide 9
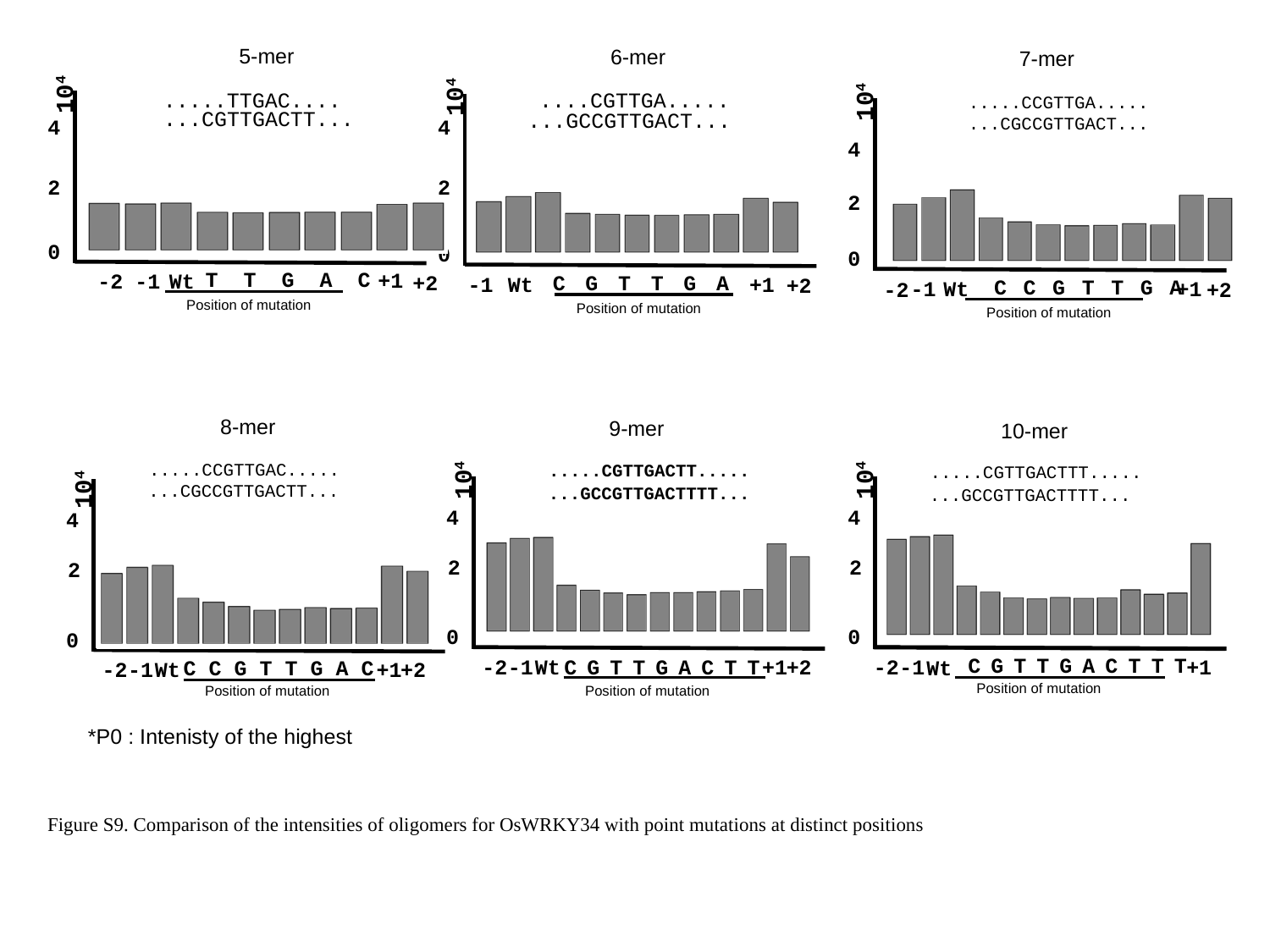

5-mer
6-mer
7-mer
104
104
....CGTTGA.....
.....TTGAC....
104
.....CCGTTGA.....
...CGTTGACTT...
...GCCGTTGACT...
...CGCCGTTGACT...
4
4
4
2
2
2
0
0
0
T T G A C
+1
-2
-1
Wt
+2
CGTTGA
-1
Wt
+1
+2
CCGTTGA
-1
Wt
+1
-2
+2
Position of mutation
Position of mutation
Position of mutation
8-mer
9-mer
10-mer
.....CCGTTGAC.....
.....CGTTGACTT.....
.....CGTTGACTTT.....
104
104
104
...CGCCGTTGACTT...
...GCCGTTGACTTTT...
...GCCGTTGACTTTT...
4
4
4
2
2
2
0
0
0
CGTTGACTTT
-2
-1
Wt
CGTTGACTT
+2
+1
+1
-2
-1
Wt
C C G T T G A C
-2
-1
Wt
+2
+1
Position of mutation
Position of mutation
Position of mutation
*P0 : Intenisty of the highest
Figure S9. Comparison of the intensities of oligomers for OsWRKY34 with point mutations at distinct positions
